# Templating Iron(III) Oxides on DNA Molecules

**DOI:** 10.3390/nano14191609

**Published:** 2024-10-07

**Authors:** Siyaka Mj Zubairu, Sulaiman O. Idris, Casmir E. Gimba, Adamu Uzairu, Andrew Houlton, Benjamin R. Horrocks

**Affiliations:** 1 Chemical Nanoscience Laboratories, School of Natural and Environmental Sciences, Bedson Building, Newcastle University, Newcastle upon Tyne NE1 7RU, UK; 2 Department of Chemistry, Ahmadu Bello University, Zaria 810211, Nigeria; soidris@abu.edu.ng (S.O.I.); cegimba@abu.edu.ng (C.E.G.); p14505@abu.edu.ng (A.U.)

**Keywords:** DNA, templating, iron oxide, goethite, haematite

## Abstract

Fe(III) oxides were prepared as free nanoparticles and on DNA templates via the precipitation of Fe(III) salts with NaOH in the presence/absence of DNA. Through control of the pH and temperature, FeOOH and Fe_2_O_3_ were synthesised. The formation of templated materials FeOOH/DNA and Fe_2_O_3_/DNA was confirmed using UV-Vis absorption and FTIR spectra. The direct optical gap of Fe_2_O_3_/DNA was estimated as 3.2 eV; the absorption by FeOOH/DNA and Fe_2_O_3_/DNA at longer wavelengths is weaker, but consistent with indirect gaps near 2 eV. X-ray photoelectron spectra confirmed the presence of Fe(III) and DNA in the templated samples. Analysis of the X-ray diffraction patterns of both templated and non-templated FeOOH and Fe_2_O_3_ demonstrated that the materials were the *α*-FeOOH and *α*-Fe_2_O_3_ polymorphs with crystallite diameters of the DNA-templated materials estimated as 7.6 nm and 6.8 nm. Transmission electron microscopy showed needle-like crystals of both FeOOH and Fe_2_O_3_, but the Fe_2_O_3_ contains some globular structures. In contrast, the morphology of FeOOH/DNA and Fe_2_O_3_/DNA consists of needle-like crystallites of the respective oxides organised into complex dendritic structures with a length on the 10 μm scale formed by the DNA molecules. Finally, scanned conductance microscopy provided evidence for the conductivity of the FeOOH/DNA after alignment via molecular combing on an Si/SiO_2_ substrate. Fe_2_O_3_/DNA did not exhibit any detectable conductivity.

## 1. Introduction

Iron (III) oxides have a range of useful chemical properties with applications in catalysis [1], chemisorption [2], as photoanodes [3,4,5] and in the case of the mixed (II/III) oxide Fe_3_O_4_ as magnetic nanoparticles [6,7,8], with uses in biomedicine [9]. Amongst the iron (III) oxides, haematite, α-Fe_2_O_3_ is a polymorph commonly found in nature and which has a long found use as a pigment [10]. It has several desirable properties; its Fe content is abundant, α-Fe_2_O_3_ is stable over a wide range of pH in aqueous media and its bulk bandgap of about 2.0 eV is convenient for the capture of sunlight [11]. Owing to its resistance to oxidation and the position of the valence band edge, α-Fe_2_O_3_ has been investigated for use as a photoanode to drive the oxygen evolution reaction [12,13]. The short carrier lifetime is a limitation of the bulk solid, but there have been attempts to address this using nanostructuring α-Fe_2_O_3_ materials [14]. Various forms of haematite including nanoparticles [15,16], nanowires [17], nanorods [18,19] and hollow nanospheres [20] have been described.

The iron (III) oxyhydroxide, α-FeOOH (Goethite), is another stable, common compound of Fe(III) with potential applications in magnetic fluids [21], in supercapacitors [22], in lithium ion batteries [23,24] and also as a photocatalyst [25,26,27]. Goethite crystals are typically needle-like [21] and nanorods have been prepared for the study of the quantum confinement effect as a function of diameter [28].

DNA-templating is a convenient method for the formation of one-dimensional nanostructures from metals [29,30,31], semiconductors [32,33,34] and conjugated polymers [35,36,37,38]. It relies on the stiffness of double-stranded DNA molecules relative to other polymers. In the case of oxide or sulphide [39,40,41] semiconductors, the metal ion is precipitated from aqueous solutions containing long DNA molecules. The DNA template has multiple binding sites for metal ions which facilitates nucleation on the template and encourages the formation of high aspect ratio nanostructures. Previous work on DNA-templated iron oxides has shown that magnetite (Fe_3_O_4_) can be precipitated on λ-DNA from mixtures of Fe(II) and Fe(III) [33]. DNA has also been used to organise pre-formed magnetite nanoparticles [42] and in a Langmuir–Blodgett monolayer to organise complex Fe_3_O_4_ nanostructures [43,44]. In view of the interest in nanostructured Fe(III) oxides, we report here the formation of α-FeOOH and α-Fe_2_O_3_ on DNA templates using the binding of aqueous Fe(III) to DNA to influence the nucleation of the oxides and investigate the morphology and spectroscopic properties of the resulting materials. The DNA-templated forms are denoted FeOOH/DNA and Fe_2_O_3_/DNA throughout.

## 2. Materials and Methods

### 2.1. Reagents and Materials

Lambda DNA (N3011, 500 μg mL^−1^, denoted as λ-DNA below) was purchased from New England Biolabs (Hitchin, UK). The phage is the heat-inducible lysogen *E. coli* l cI857 S7. λ-DNA is obtained via phenol extraction and then dialysed against 10 mM Tris-HCl (pH 8.0) + 1 mM EDTA. λ-DNA comprises 48,502 base pairs [45]. For experiments requiring larger sample sizes, DNA (Sodium salt) from Herring Testes (HT-DNA) (type XIV) was used (Sigma-Aldrich).

The solvents isopropanol, acetone, methanol and ethanol were purchased from Fisher Scientific Ltd (Loughborough, UK) while chloroform was obtained from Sigma-Aldrich (Gillingham, UK); all were used as received (>99% purity). Fe(NO_3_)_3_·9H_2_O (>97%), FeCl_3_·6H_2_O (>98%), NaOH pellets (98–100%), H_2_O_2_ 30 vol, H_2_SO_4_ (conc) and Me_3_SiCl (CTMS) were obtained from Sigma-Aldrich (Gillingham, UK).

Aqueous solutions were prepared with deionised water (nominal 18.2 M Ω cm resistivity) from a NANOpure^TM^ Diamond UV ultrapure water system equipped with a diamond RO reverse osmosis system (Barnstead International, Dubuque, IA, USA).

n-Si 〈111〉 wafers that were phosphorous-doped, 525±25 μm thickness, 1–12 Ω cm resistivity and single-side polished were used as supports for atomic force microscopy (AFM) imaging, infrared spectroscopy (FTIR), X-ray diffraction (XRD) and X-ray photoelectron spectroscopy (XPS). n-Si 〈100〉 wafers that were arsenic doped, ≤0.005Ω cm resistivity, with a 200 nm thick SiO_2_ layer and double-sided polished were used for scanned conductance microscopy (SCM). All Si wafers were purchased from PI-KEM Ltd. (Tamworth, UK).

### 2.2. Preparation of FeOOH/DNA

FeOOH was templated on DNA using aqueous Fe(NO_3_)_3_ as the source of Fe(III) and NaOH(aq) as precipitant.

For the microscopy studies (TEM/AFM/SCM) and photoelectron spectroscopy (XPS): Samples of DNA/FeOOH were prepared by mixing 12 μL of Fe(NO_3_)_3_·9H_2_O (3 mM) with 6 μL of 200 μg mL^−1^λ-DNA (diluted from 500 μg mL^−1^ stock) in an Eppendorf tube. NaOH (1.7 mM) was added slowly (about 6 μL) with stirring using a micropipet until the solution pH was about 12. The solution was incubated for 6 h at room temperature followed by ageing for 48 h at 20 °C.

For the spectroscopic studies (FTIR, UV-Vis, XRD), where larger samples were required: Fe(NO_3_)_3_·9H_2_O (3 mM, 0.5 mL) was added to HT-DNA (200 μg mL^−1^, 0.25 mL) with stirring for 30 min. Then NaOH (55 mM) was added slowly (about 0.5 mL) via a micropipet until the solution pH was about 12 and the sample left to age for 48 h at 20 °C.

### 2.3. Preparation of Fe_2_O_3_/DNA

Fe_2_O_3_ was templated on DNA using aqueous FeCl_3_ as the source of Fe(III) and NaOH(aq) as precipitant.

The preparation followed that for FeOOH/DNA above except (i) FeCl_3_·6H_2_O was used in place of Fe(NO_3_)_3_·9H_2_O, and (ii) after addition of NaOH, the sample was incubated at 60 °C for 1 h. Again, λ-DNA was used for preparation of samples for microscopy and HT-DNA was used to prepare larger samples for spectroscopic characterisation (quantities given in Section 2.2).

### 2.4. Preparation of Non-Templated FeOOH Nanoparticles

Samples of FeOOH nanoparticles for comparison with templated FeOOH/DNA were prepared using slow hydrolysis. NaOH (1.5 mM) was added to an aqueous solution of Fe(NO_3_)_3_·9H_2_O (200 μL, 3.5 mM) at room temperature with stirring. A dark brown precipitate was formed; the colour changed on standing to the characteristic reddish brown of Fe_2_O_3_. The pH of the solution was adjusted to 12.9 and filtered to collect the precipitate. After washing with water until the filtrate was clear, the product was dispersed in 1.5 mM NaOH and allowed to stand. A bright yellow precipitate was observed after 1 day. The precipitate was redispersed and transferred into a 40 mL Teflon autoclave and maintained at 18 °C for 24 h and then at 80 °C for 10 h. After cooling, the yellow precipitate was collected via centrifugation and washed repeatedly with ethanol and deionised water. After drying in a vacuum at 100 °C for 10 h, the prepared nanoparticles were purified through suspending in 2.0 mL of HCl(aq) (10 mM). The FeOOH was collected via centrifugation and calcined in air at 150 °C.

### 2.5. Preparation of Non-Templated Fe_2_O_3_

Samples of Fe_2_O_3_ nanoparticles for comparison with Fe_2_O_3_/DNA were prepared using a two-step reaction process involving hydrolysis and then calcination to produce Fe_2_O_3_. In this case, FeCl_3_·6H_2_O was used as the Fe(III) precursor. Sufficient NaOH (3.6 M) was added to FeCl_3_·6H_2_O (0.50 M, 4 mL) aqueous solution to achieve a pH of about 12. A further 10 mL of nanopure water was added and the mixture maintained at 140 °C overnight. The fine red precipitate was collected via filtration and washed repeatedly with methanol and deionised water until the filtrate was free of acid; then, it was dried at 100 °C for one day. The resultant product was heated in air from room temperature to 500 °C at a heating rate of 2 °C min^−1^ and maintained at 400 °C for 2 h.

### 2.6. Si Substrate Preparation

n-Si 〈111〉 wafers were cut into approximately 1 cm^2^ pieces using a diamond pencil and cleaned with acetone and isopropanol. The chips were treated with fresh piranha solution (H_2_SO_4_:H_2_O_2_ 4:1) for 45 min. The chips were then rinsed with deionised water, blown dry in a stream of N_2_ and dried in an oven for 8 min. The polished surface was silanised by placing it over a glass vial containing 0.1 mL chlorotrimethylsilane and allowing the surface to react with the vapour for 10 min. This treatment produces partially silanised surfaces with a static contact angle of about 70 °C.

Substrates for scanned conductance microscopy were prepared on n-Si 〈100〉 (1 cm^2^) chips with a 200 nm thick SiO_2_ layer. These were also treated with piranha solution, washed with deionised water, dried and silanised with chlorotrimethylsilane.

The partial silanisation treatment facilitates alignment of the nanowires by reducing their adhesion to the Si substrate and this was carried out using molecular combing [46].

### 2.7. Optical Absorption Spectroscopy

Optical absorption spectra were recorded in transmission mode on a Cary 100 Bio UV-Visible spectrophotometer (Varian, Palo Alto, CA, USA) at room temperature (about 293 K) over the wavelength range 200–800 nm. A quartz microcuvette of pathlength 1 cm was used for the sample, as prepared in Section 2.2. The samples are coloured, but non-turbid aqueous dispersions were recorded as judged by the lack of a long wavelength tail in the lowest energy region of the measured spectrum. The blank was deionised water. Tauc plots were calculated directly from the measured absorbances *A* by plotting ${\left(Ah\nu \right)}^{n}$ against photon energy (eV) for n=2 (direct transition) or n=1/2 (indirect transition).

### 2.8. FTIR

Infrared spectra were recorded at a resolution of 4 cm^−1^ in transmission mode on a Varian 800 FTIR spectrometer. A total of 124 scans were co-added and averaged. The sample (70 μL) was drop-cast on a Si 〈111〉 chip and dried in vacuo. A clean Si chip was used as the blank. The spectra were baselined using the instrument-supplied software.

### 2.9. Photoelectron Spectroscopy (XPS)

Samples were deposited on Si chips 〈111〉 and mounted on Cu stubs with carbon tape. 5 μL of solution was deposited on a clean Si wafer and left to dry at room temperature in a laminar flow hood to minimise contamination. The sample was not washed on the substrate owing to its small size. XPS measurements were taken on a Kα spectrometer (ThermoFisher Scientific, Waltham, MA, USA) equipped with an Al Kα X-ray source (1486.6 eV), an electron flood gun and a operating power of ≃100 W (15 kV, 7 mA) at a take-off angle of 90°. Binding energies were calibrated by setting the lowest binding energy component of the C 1s spectrum to 284.8 eV [47]. The 1s spectra for C, N, O and P were modelled using a simple linear background and singlet functions of the Gaussian–Lorentzian sum form (50:50). In the case of Fe 2p spectra, a Shirley background was employed [48] and the spectra were modelled with Gaussian–Lorentzian (50:50) sum functions in which the relative areas of the $2p_{1/2}$ and $2p_{3/2}$ peaks were constrained to a 1:2 ratio during the least-squares fitting. Depth profiles were obtained using an EX06 ion source (ThermoFisher Scientific) at 200 eV.

### 2.10. Powder X-ray Diffraction

Samples for powder X-ray diffraction were drop-cast on Si chips and allowed to dry in a laminar flow hood to minimise contamination. The quantities of FeOOH/DNA and Fe_2_O_3_/DNA that were used are given in the preparation sections. The diffraction patterns were recorded on a Bruker D2 phaser bench top diffractometer with a Cu Kα radiation source (λ = 1.54178 Å) at a scan rate of 1 deg·s^−1^ over the range $2<2\theta <60^{\circ }$.

Crystallite sizes were calculated using the Scherrer equation. The data were modelled using a sum of Gaussian functions centred at the 2θ values of the literature patterns for α-FeOOH and α-Fe_2_O_3_. The peak widths and amplitudes were estimated using the method of least squares. The background was modelled using a linear function and a single, broad Gaussian function.

### 2.11. Atomic Force and Scanned Conductance Microscopy

Samples were prepared via the drop-casting 3 μL of the templated DNA solution onto the edge of freshly silanised Si chips. The droplet was then dragged across the surface (’molecular combing’ [46]) and allowed to dry in a laminar flow hood for 7–10 min under a stream of N_2_.

AFM images were acquired in air on a Nanoscope V Dimension AFM system (Bruker, Billerica, MA, USA) in Tapping mode using TESP7 (n-doped Si cantilevers, with a resonance frequency of 230–280 kHz and a force constant range of 20–80 Nm^−1^ AFM probes (Bruker). Vibrational noise was reduced using an acoustic isolation enclosure (Bruker). Background subtraction and height profile measurements were performed using the open-source image analysis program Gwyddion version 2.58 [49].

Scanning conductance microscopy (SCM) measurements were carried out in air using MESP probes (n-doped Si cantilevers) with a metallic Co/Cr coating, a resonance frequency of 60–100 kHz and a spring constant of 1–5 N m^−1^. SCM measurements were conducted using a dual-pass mode. The sample topography was recorded in tapping mode in the first pass. In the second pass, the tip retraces the topography at a specified lift height (here 50 nm) and the phase of the tip motion with respect to the driving signal was recorded. A dc potential was applied between the sample and the tip at a value chosen between −7 V and +7 V.

### 2.12. Electron Microscopy

Samples for transmission electron microscopy were prepared by placing a drop of solution onto the TEM grid (holey carbon-coated Cu grid (400 mesh), Agar Scientific, Rotherham, UK) and allowed to dry slowly at room temperature. The grids were examined using a Philips CM 100 Compustage (FEI) transmission electron microscope and digital images were collected using an AMT CCD camera (Deben, Suffolk, UK).

## 3. Results and Discussion

The preparation of Fe(III) oxides on a DNA template involves the initial association of Fe^3+^ ions with the DNA followed by reaction with OH^−^ to precipitate the oxide/oxyhydroxide. Fe(III) oxides/oxyhydroxides exist in various forms, including FeOOH and Fe_2_O_3_ and their polymorphs. We first characterise the chemical composition of both free, non-templated nanoparticles and the DNA-templated materials. Through the control of the reaction conditions (pH, ageing time), we show that both FeOOH and Fe_2_O_3_ can be prepared on DNA templates and that they exhibit an unusual dendritic, brush-like morphology. Finally, we demonstrate that the FeOOH/DNA structures are conductive, but the Fe_2_O_3_/DNA structures are not.

### 3.1. Chemical Characterisation

#### 3.1.1. Fourier Transform Infrared Spectroscopy

**Figure 1:**
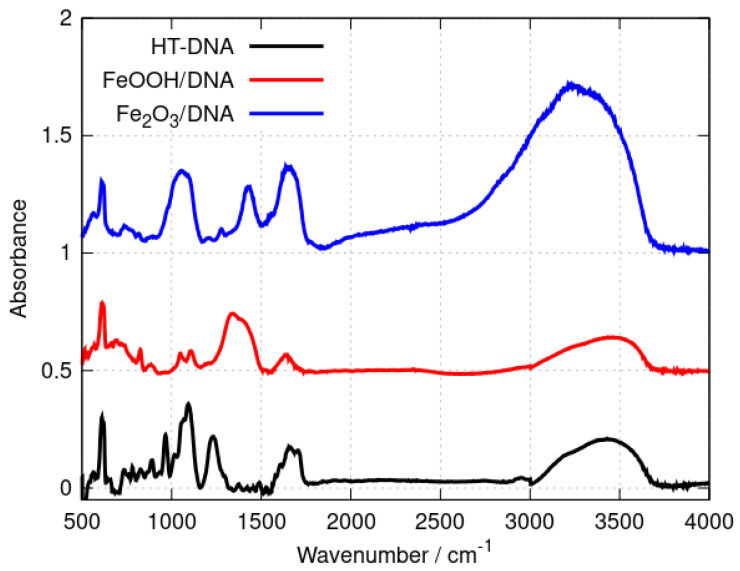
FTIR spectra of (black) HT−DNA, (red) FeOOH/DNA and (blue) Fe_2_O_3_/DNA. The absorbances were scaled to be equal for the sharp feature at 611 cm^−1^ and offset on the y-axis for clarity.

FTIR spectroscopy provides a convenient method to distinguish DNA-templated materials from simple mixtures of DNA and nanoparticles of the material. Shifts in the band positions of DNA vibration modes demonstrate the interaction of the material with the template. Figure 1 shows the spectra of the DNA, Fe_2_O_3_/DNA and FeOOH/DNA samples drop-cast onto Si chips.

The broad band in the range 3000–3500 cm^−1^ includes O-H and N-H stretching vibrations and is present in all three samples. This band contains contributions from adsorbed water as well as the N-H modes of the nucleobases. Qualitative differences between the three spectra are observed in the range 1300–1750 cm^−1^ where nucleobase vibrations and C=O stretches appear [50,51,52] as well as bending modes of adsorbed water. However, an examination of the symmetric and asymmetric P-O stretching region (1100–1250 cm^−1^) provides clear evidence of the interaction of the iron oxides with the phosphate backbone of the DNA. Bare HT-DNA shows a peak assigned to the symmetric stretch near 1094 cm^−1^ and a peak assigned to the corresponding asymmetric stretch at 1233 cm^−1^. In contrast, the asymmetric P-O stretching mode is either broadened (Fe_2_O_3_/DNA) or absent or so strongly shifted so that it is part of a broad feature near 1339 cm^−1^ in FeOOH/DNA. The data demonstrate the intimate interaction of the iron oxides with the DNA, but they are not sufficient to characterise the oxides and we therefore employed X-ray photoelectron spectroscopy and X-ray diffraction for definitive assignment of the templated oxide structures.

#### 3.1.2. X-ray Photoelectron Spectroscopy

**Figure 2:**
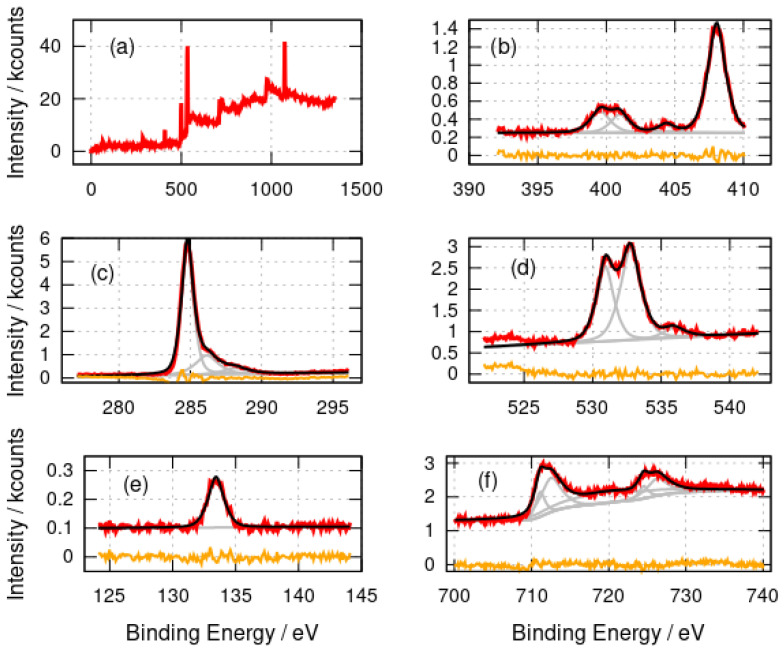
X-ray photoelectron spectra of α-FeOOH/DNA. (**a**) Survey spectrum; (**b**) N 1s spectrum; (**c**) C 1s spectrum; (**d**) O 1s spectrum; (**e**) P 2p spectrum and (**f**) Fe 2p spectrum. The experimental data is shown in red, the fits are shown in black and the individual fitting components are shown in gray. The orange lines are the residuals after fitting.

Figure 2 shows the survey and high-resolution photoelectron spectra of α-FeOOH/DNA. The elements expected (C,N,O,P, Fe) were observed as well as Na (countercation in DNA) and Cl (present as Cl^−^ in the DNA buffer). The N 1s spectrum was fitted with three major components at binding energies of 399.6 eV, 401.0 eV and 408.0 eV and a minor component at 404.4 eV. The components at 399.6 eV (-NH-, -N=) and 401.0 eV (amine, -NH_2_) are assigned to the nitrogen atoms in the DNA bases [53]. The peak at 408.0 eV is simply residual nitrate from the preparation, which employed Fe(NO_3_)_3_, and matches the binding energy of nitrate in NH_4_NO_3_ [54]. The C 1s spectrum also has the form expected for DNA with a large component at 284.8 eV (C-C) and two smaller components at 286.2 eV and 288.2 eV which we assign to C-O and C=O functionalities in DNA. The O 1s spectra have two major components at 530.9 eV and 532.8 eV which are assigned to an unresolved combination of O atoms in DNA and phosphate [55] or nitrate groups [56]. The 530.9 eV component likely also contains a contribution from O atoms in iron oxides [57,58]; however, this cannot be resolved definitively from the other contributions. The small feature at 535.9 eV may be physisorbed water [59]. Similar spectra with two components resolved in the O 1s region have been reported for DNA films [60]. The P 2p spectrum shows a single peak at 133.4 eV which is typical of phosphate in DNA [60].

**Figure 3:**
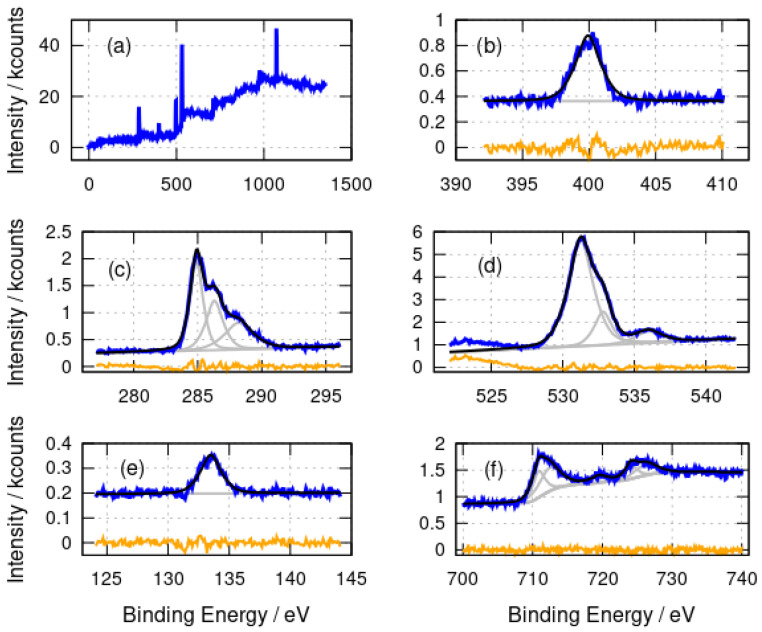
X-ray photoelectron spectra of α-Fe_2_O_3_/DNA. (**a**) Survey spectrum; (**b**) N 1s spectrum; (**c**) C 1s spectrum; (**d**) O 1s spectrum; (**e**) P 2p spectrum and (**f**) Fe 2p spectrum. The experimental data is shown in blue, the fits are shown in black and the individual fitting components are shown in gray. The orange lines are the residuals after fitting.

A very similar assignment of the N 1s, C 1s, O 1s and P 2p spectra (Figure 3) for α-Fe_2_O_3_/DNA can be made. Nitrate is absent from the Fe_2_O_3_ preparation and a peak at 408 eV is not observed in the N 1s spectrum (Figure 3b). The signal-to-noise ratio is not sufficient to resolve the components from different N chemical environments in α-Fe_2_O_3_/DNA, but a single band centred at 399.9 eV is observed. The C 1s spectra (Figure 3c) show the same components at 284.9 eV, 286.2 eV and 288.2 eV as in α-FeOOH/DNA and the O 1s spectra also have similar components at 531.3 eV and 532.8 eV, but the higher binding energy peak is smaller than in α-FeOOH/DNA, presumably because the nitrate was not used in the preparation of α-Fe_2_O_3_/DNA. Finally, there is the expected P 2p peak at 133.5 eV for DNA.

The Fe 2p spectra provide more information on the nature of the templated material rather than the DNA template. The binding energies of the fitted components are collected in Table 1.

The main Fe 2p peaks are broad and were not well-fitted by simple Gaussian–Lorentzian functions; therefore, both the $2p^{3/2}$ and $2p^{1/2}$ peaks were fitted using two such functions. This is understandable because of the complex nature of these features [61]. The lower binding energy component models the region of the peak and the higher binding energy component is necessary to model the shape of the band to the right of the peak. The binding energies for the $2p^{3/2}$ peaks at 711.1 eV (α-FeOOH/DNA) and 710.9 eV (α-Fe_2_O_3_/DNA) are in agreement with the literature values for FeOOH and Fe_2_O_3_ [58,62] and with α-Fe_2_O_3_ films on alumina substrates [63]. The binding energies observed are consistent with Fe(III) and not Fe(II) or Fe(0). The broad shake-up satellite is clearly resolved at 719.7 eV which is also characteristic of Fe(III). The corresponding satellite of the $2p^{1/2}$ peak is too weak to be clearly resolved. An Fe(II) satellite near 716 eV is not observed and there is no evidence of a shoulder on the low binding energy side of the $2p^{3/2}$ peak which would be seen in Fe_3_O_4_.

**Figure 4:**
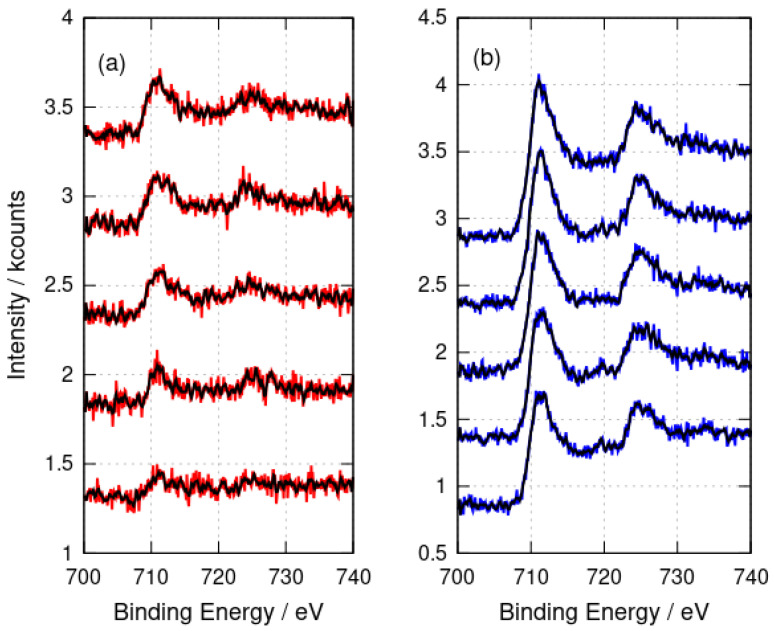
X-ray photoelectron spectra after argon-ion etching for 0 s, 400 s, 800 s, 1200 s and 1600 s. The spectra are stacked by an increment of 1 kcount on the y-axis for each 400 s of etching time. (**a**) α-FeOOH/DNA (red) and (**b**) α-Fe_2_O_3_/DNA (blue). The black lines show the result of applying a 9-point quadratic Savitzky–Golay filter to the raw data.

XPS analyses the sample surface and, in principle, some variation with depth inside the sample might be observed. However, the etching of the samples with Ar^+^ for times up to 1600 s showed no change in the Fe 2p peak positions (Figure 4) which would indicate the presence of Fe(II). In conclusion, the XPS data are consistent with DNA structures coated with Fe(III) oxides; however, core level XPS is not well suited to distinguish FeOOH from Fe_2_O_3_ [62].

#### 3.1.3. Powder X-ray Diffraction

**Figure 5:**
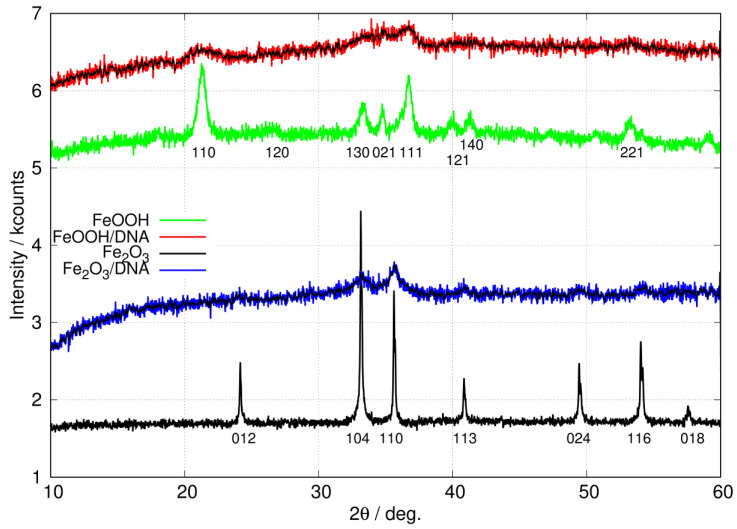
XRD patterns of free, non-templated nanoparticles of FeOOH (green) and Fe_2_O_3_ (black) compared to patterns for DNA-templated FeOOH/DNA (red) and Fe_2_O_3_/DNA (blue). The black lines superimposed on the FeOOH/DNA and Fe_2_O_3_/DNA patterns show the result of applying a 9-point quadratic Savitzky–Golay filter to the raw data. The patterns are offset on the y-axis for clarity.

Figure 5 shows the X-ray diffraction (XRD) patterns of the non-templated nanoparticles of FeOOH and Fe_2_O_3_ as well as the patterns for the DNA-templated materials, FeOOH/DNA and Fe_2_O_3_/DNA. The major peaks for non-templated FeOOH and Fe_2_O_3_ nanoparticles are collected in Table 2.

The XRD pattern of non-templated FeOOH nanoparticles is in good agreement with previous reports for α-FeOOH [64,65]. In particular, the peak at 2θ=21.3 degrees is present in α-FeOOH but not in the β, γ or δ polymorphs. Further, these polymorphs also show peaks assigned to (110), (200) or (001) reflections in the range 10<2θ<20 degrees which we do not observe [65]. Equally, the XRD pattern of non-templated Fe_2_O_3_ nanoparticles matches the literature reports on α-Fe_2_O_3_ [66,67,68,69] and is free of peaks which might be assigned to FeOOH or β-Fe_2_O_3_ [70]. Finally, there were no detectable peaks matching Fe_3_O_4_ [71,72,73,74] or γ-Fe_2_O_3_ [70,75].

**Figure 6:**
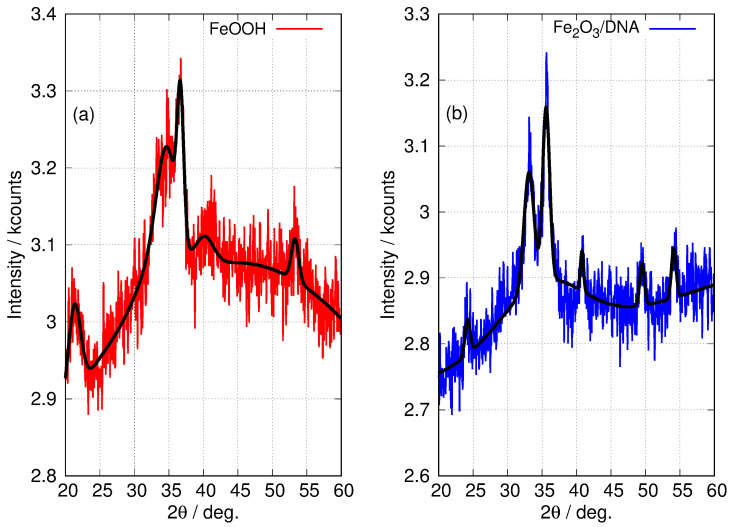
Fitted XRD patterns of DNA-templated Fe(III) oxides. (**a**) FeOOH/DNA (red) and (**b**) Fe_2_O_3_/DNA (blue). The black lines superimposed on the patterns show the result of a least squares fit to a sum of Gaussian functions centred at the positions of the 6 most prominent peaks in α-FeOOH and α-Fe_2_O_3_ (see Table 2). Only the widths and intensities of these peaks were floated to obtain the fit. A combination of a linear function and a Gaussian function of large width was used to model the background.

The patterns for the templated materials, FeOOH/DNA and Fe_2_O_3_/DNA, have poorer signal-to-noise ratios owing to the relatively small sample sizes that can be prepared. However, the peaks that are detectable match those in the non-templated nanoparticle samples and we assign these patterns to α-FeOOH/DNA and α-Fe_2_O_3_/DNA. Figure 6 shows the powder patterns of the DNA-templated samples in more detail along with the fit of a regression model based on a sum of Gaussians. In the case of FeOOH/DNA the (140) reflection is not distinguished from the (121) reflection because of the signal-to-noise ratio. However, all the other expected reflections are clearly observed. Based on the Scherrer analysis for the most prominent peak at $2\theta =36.7^{\circ }$ for the (111) reflection, we estimate a crystallite diameter of 7.6 nm. In the case of Fe_2_O_3_/DNA, the (018) reflection is not detectable above the noise and is not fitted. All the other expected reflections are clearly observed. Based on the Scherrer analysis for the most prominent peak at $2\theta =35.6^{\circ }$ for the (110) reflection, we estimate a crystallite diameter of 6.8 nm.

### 3.2. Optical Spectra and the Bandgap of DNA-Templated FeOOH and Fe_2_O_3_

#### Ultra-Violet Absorption Spectroscopy

UV-Vis absorption spectroscopy provides a straightforward means to demonstrate the formation of iron oxides and the presence of DNA in the preparations.

**Figure 7:**
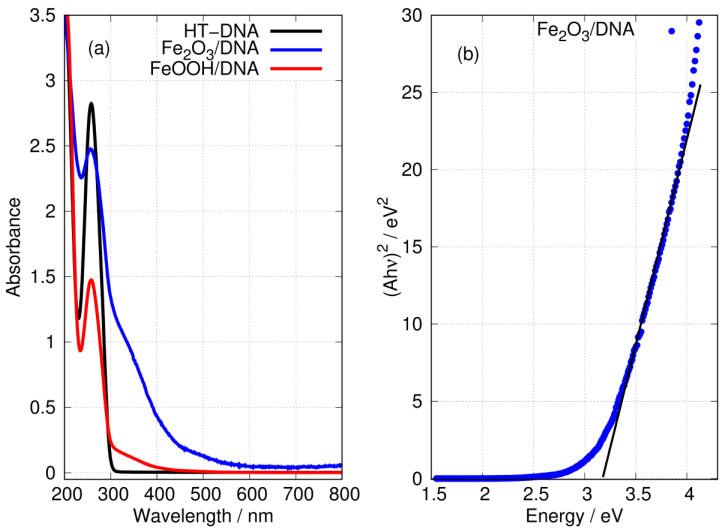
(**a**) UV-Vis absorption spectra in aqueous solution of (black) HT-DNA, (blue) Fe_2_O_3_/DNA and (red) FeOOH/DNA. The absorbances of Fe_2_O_3_/DNA and FeOOH/DNA were scaled to equal that of HT-DNA at 200 nm. (**b**) Tauc plot (direct) of the data for Fe_2_O_3_/DNA showing $E_{opt,direct}≃3.2$ eV.

Figure 7 shows the characteristic absorption band of DNA at a wavelength of 260 nm. The preparations of FeOOH/DNA and Fe_2_O_3_/DNA also show this band, but its intensity is diminished. However, the spectra of DNA-templated Fe(III) oxides both show substantial absorption tails in the 300<λ<600 nm region where DNA itself has no absorption. The absorbance of FeOOH/DNA decreases monotonically with a wavelength below 300 nm with no marked structure; this is consistent with reports for FeOOH nanoparticles [76]. The absorption spectrum of Fe_2_O_3_/DNA is more intense and extends to wavelengths approaching 600 nm, and there are two distinct regions: (i) a weak shoulder in the region 450<λ<600 nm and (ii) a more pronounced shoulder in the region 300<λ<450 nm. The general appearance of the Fe_2_O_3_/DNA spectrum is also consistent with reports on Fe_2_O_3_ nanoparticles [66,77]. The electronic structure of Fe_2_O_3_ is considered to be that of a Mott insulator and has been studied computationally using the GW method [78]. The optical gap near 2.1 eV is considered indirect [79] and this corresponds to the weak absorption below 600 nm. In thin films composed of Fe_2_O_3_ and NiO, the data below 450 nm have been analysed as a direct transition in Fe_2_O_3_ with a gap in the range 2.99–3.35 eV [80]. We obtained a similar estimate of 3.2 eV for the direct gap in Fe_2_O_3_/DNA in Figure 7b.

**Figure 8:**
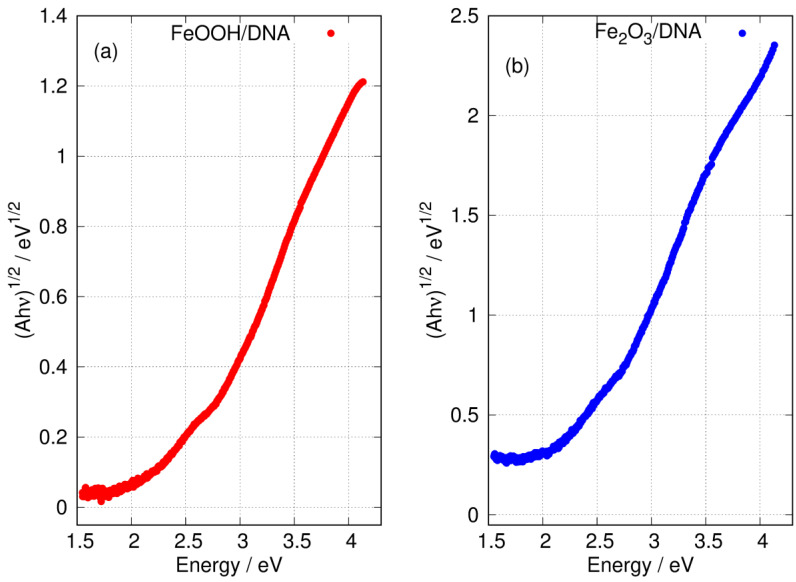
Tauc plots of the low energy region of the absorption spectra with exponent appropriate for an indirect gap. (**a**) FeOOH/DNA and (**b**) Fe_2_O_3_/DNA.

Figure 8 shows the Tauc plot of the longer wavelength region for both FeOOH/DNA and Fe_2_O_3_/DNA according to the exponent appropriate for an indirect allowed gap. In this region, the plot is not clearly linear and a determination of $E_{opt,indirect}$ may be affected by states related to disorder or defects [81]. However, the onset of absorption clearly occurs near 2 eV as expected for FeOOH [27,28] and Fe_2_O_3_ [79].

### 3.3. Morphology and Conductivity of DNA-Templated FeOOH and Fe_2_O_3_

#### Transmission Electron Microscopy

**Figure 9:**
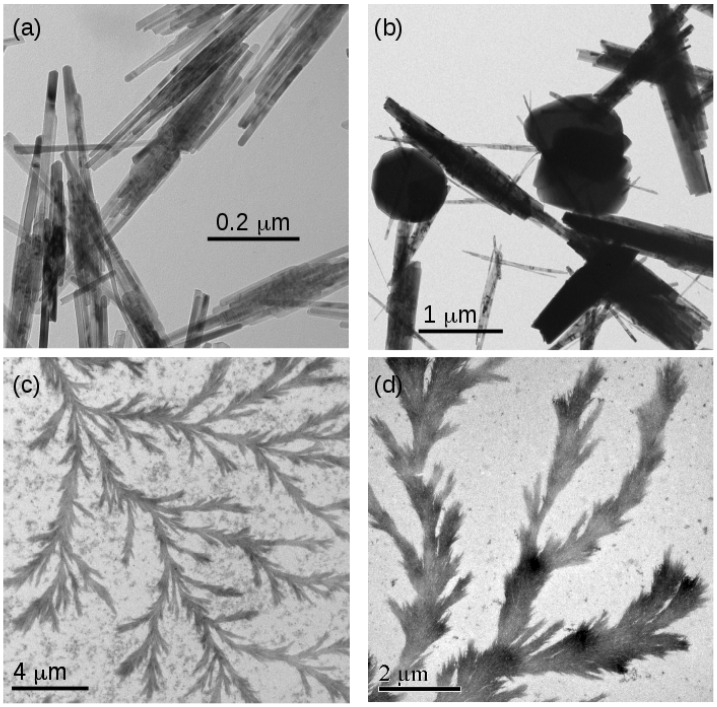
TEM images of samples of non-templated and templated FeOOH and Fe_2_O_3_. (**a**) Non-templated α-FeOOH; (**b**) non-templated α-Fe_2_O_3_; (**c**) templated FeOOH/DNA and (**d**) templated Fe_2_O_3_/DNA.

The morphology of the FeOOH and Fe_2_O_3_ samples was investigated using transmission electron microscopy. Figure 9 shows typical images of templated and non-templated material. Figure 9a,b show non-templated FeOOH and Fe_2_O_3_, respectively. The characteristic needle-like crystals of α−FeOOH are observed [21]. The diameter of the needles is approximately consistent with the estimates of the crystallite diameter from the Scherrer analysis in Table 2. In the case of Fe_2_O_3_, some globular particles were also found. These features are much larger than the crystallite sizes for Fe_2_O_3_ determined using XRD analysis, which suggests polycrystallinity.

In contrast, the DNA-templated forms of both FeOOH and Fe_2_O_3_ are dendritic and organised into larger structures using the DNA template (Figure 9c,d). The TEM images clearly show a thickness larger than the diameter of bare DNA (2 nm) and also much wider than the iron oxide crystallite diameters determined using XRD analysis (Figure 6). This indicates a complex internal structure in which the small crystallites are organised into long structures by the DNA molecules. The strong image contrast is expected for metal oxides and indicates that these structures comprise FeOOH and Fe_2_O_3_ rather than solely organic material.

### 3.4. Atomic Force and Scanned Conductance Microscopy

**Figure 10:**
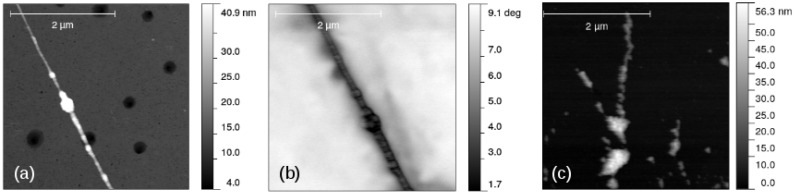
Images of α-FeOOH/DNA and α-Fe_2_O_3_/DNA aligned using molecular combing on a Si/SiO_2_ substrate. (**a**) AFM image of α-FeOOH/DNA; (**b**) SCM phase image of α-FeOOH/DNA (lift height 50 nm, bias voltage = 6 V) and (**c**) AFM image of α-Fe_2_O_3_/DNA.

Aligned one-dimensional α-FeOOH/DNA and α-Fe_2_O_3_/DNA structures on Si/SiO_2_ surfaces were prepared using molecular combing in which a droplet of the sample was dragged across a partially silanised surface [46]. These structures were imaged using atomic force microscopy and the examples are shown in Figure 10. The dendritic structures observed in TEM (Figure 9) are not evident in this case, an observation which we attribute to the forces on the structures arising from fluid flow and interaction with the substrate during combing. A comparison of Figure 9c and Figure 10a suggests that the branches of the structure seen in electron microscopy are wrapped around each other and compacted during the molecular combing procedure used in sample preparation for probe microscopy.

Nevertheless, the conductivity of these structures was assessed in a qualitative manner using scanned conductance microscopy. In this two-pass technique, the first pass records the topography and in the second pass the metallised tip is lifted a fixed height above the topography; the phase of the tip oscillation with respect to the signal driving the tapping motion is recorded as the tip returns back along the line scanned in the first pass. The variation in the phase angle depends on the capacitive coupling between the tip and sample as the tip passes over the nanostructure that is being imaged. In the case of an insulating nanostructure, the phase shift is always positive with respect to the background because the dc potential applied between the tip and substrate only induces polarisation in the immediate vicinity of the tip. However, for conductive structures, the bias potential induces a polarisation of a region determined by the RC time constant of the structure compared to the oscillation frequency (ω) [82]. For nanostructures, the capacitance is very small and therefore it is often the case that ωRC<<1. In this case, the phase shift as the tip crosses the nanowire is negative and arises because the second derivative of the energy stored in the tip/sample capacitance with respect to tip displacement is equivalent to an additional contribution to the force constant of the cantilever. The outcome is a phase shift Δϕ proportional to the square of the applied dc bias potential *V* given by Equation (1) [83].

(1) $$\tan\left(\Delta \phi \right)=\frac{Q}{2k}V^{2}\left[\frac{2\pi R^{2}\epsilon_{0}}{{\left(h+\frac{t}{\epsilon_{ox}}\right)}^{3}}-\frac{2\pi L^{2}\epsilon_{0}}{{\left(h+\frac{t}{\epsilon_{ox}}+\frac{d}{\epsilon }\right)}^{3}}\right]$$

where *d* is the nanowire thickness, *t* is the thickness of the dielectric layer (SiO_2_) on the substrate and ϵ and $\epsilon_{ox}$ are the corresponding relative permittivities. In this model, the phase shift Δϕ depends on the applied tip bias *V* and the ratio of the quality factor to the cantilever spring constant $\frac{Q}{k}$. *R* is the tip radius and *L* is the size of the region that is polarised by the tip; if the structure is insulating L≃R and Δϕ>0, but if the structure is sufficiently conductive, L≫R and Δϕ<0. Equation (1) provides a sensitive, non-contact test for conductivity in nanostructures and also shows that the conductance effect can be distinguished from surface charge effects because of the quadratic (as opposed to linear) variation in the dc bias *V* applied between sample and tip.

**Figure 11:**
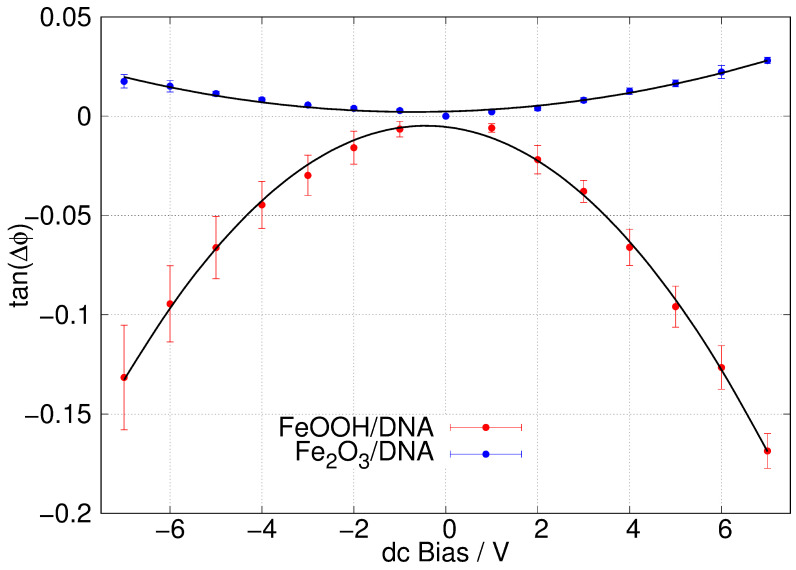
Plots of tanϕ against dc bias voltage (ϕ = SCM phase angle). The samples were α-FeOOH/DNA (red) and α-Fe_2_O_3_/DNA (blue) aligned via molecular combing on a Si/SiO_2_ substrate. The lift height was 50 nm. The error bars shown are standard deviations based on N = 5 (red) and N = 4 (blue) measurements.

Figure 10b shows a typical SCM phase image of α-FeOOH/DNA on a Si/SiO_2_ substrate. The change in phase as the tip crosses the structure, running NW-SE in the image, is clearly negative which indicates some conductivity. These measurements have the advantage of being insensitive to contact resistance, although they do not enable a quantitative estimate of the conductivity when ωRC<<1. Figure 11 shows the corresponding plots of tan(Δϕ) against *V* with least squares fits of Equation (1) shown as black lines. The data show (i) the SCM conductance effect, rather than the fields from the trapped charges (electric force microscopy) dominating and that (ii) α-FeOOH/DNA is detectably conductive, but α-Fe_2_O_3_/DNA is not. There is no reason to expect that FeOOH is inherently a better conductor than Fe_2_O_3_. In fact, both are known to be weakly semiconducting [84,85], which is sufficient for observation by SCM. However, the lack of conductivity of α-Fe_2_O_3_/DNA in our work may be a result of a subtle difference in morphology that leads to more breaks in the conduction pathway.

## 4. Conclusions

A simple hydrolysis of Fe(III) salts using hydroxide ions in the presence of DNA results in the DNA templating of the oxide α-Fe_2_O_3_/DNA or the oxyhydroxide α-FeOOH/DNA. The α polymorphs were confirmed using X-ray diffraction and the Fe(III) oxidation state using X-ray photoelectron spectroscopy. The Scherrer analysis of the crystallite diameters indicated values of 7.6 nm for α-FeOOH/DNA and 6.8 nm for α-Fe_2_O_3_/DNA. The optical gaps of the materials are consistent with previous reports of an indirect gap near 2 eV and, a direct gap at higher energies, which we measured as 3.2 eV for Fe_2_O_3_/DNA. That for FeOOH/DNA was partly obscured by the onset of the absorption in the DNA bases.

The morphology of the α-FeOOH/DNA and α-Fe_2_O_3_/DNA shows striking dendritic features in which the needle-like crystals of the inorganic component are organised by the DNA molecules into larger structures of at least 10 μm length. Interestingly, we find that α-FeOOH/DNA samples are conductive using the non-contact scanned conductance microscopy technique, but that α-Fe_2_O_3_/DNA samples are not.

## Figures and Tables

**Table 1:** Binding energies of the fitted components (eV) in the Fe 2p spectra of α-FeOOH/DNA and α-Fe_2_O_3_/DNA. “Sat.” denotes the shake-up satellite associated with the $2p^{3/2}$ peak.

	Binding Energies/eV				
**Sample**	$2p^{3/2}$		**Sat.**	$2p^{1/2}$	
α-FeOOH/DNA	711.1	712.5	719.7	724.5	726.3
α-Fe_2_O_3_/DNA	710.9	712.5	719.6	724.1	726.3

**Table 2:** Major peaks (2θ/degrees) observed in the XRD patterns of non-templated FeOOH and Fe_2_O_3_ nanoparticles in Figure 5. Reflections labelled * show the peak positions used to fit the XRD patterns of the templated materials FeOOH/DNA and Fe_2_O_3_/DNA in Figure 6. ^‡^ Determined via analysis of the 7 assigned peaks for the non-templated FeOOH and Fe_2_O_3_.

Sample	2θ/Degrees and Assignment (hkl)							^‡^ $D_{\mathrm{Scherrer}}$/nm
FeOOH	21.3	33.2	34.7	36.7	40.1	41.4	53.3	11±2.6
	(110) *	(130) *	(021) *	(111) *	(121) *	(140)	(221) *	
Fe_2_O_3_	24.2	33.1	35.6	40.8	49.5	54.1	57.6	31±5.3
	(012) *	(104) *	(110) *	(113) *	(024) *	(116) *	(018)	

## Data Availability

The original contributions presented in the study are included in the article/Supplementary Materials; further inquiries can be directed to the corresponding authors.

## Supplementary Materials

The following supporting information can be downloaded at: https://www.mdpi.com/article/10.3390/nano14191609/s1, File S1: Dataset.
